# Supplementary material for: Associations between Second-Hand Tobacco Smoke Exposure and Cardiorespiratory Fitness, Physical Activity, and Respiratory Health in Children
Source: Int J Environ Res Public Health. 2021 Oct 30;18(21):11445. doi: 10.3390/ijerph182111445 (PMC8582797; doi:10.3390/ijerph182111445)
Supplement: Supplementary file 1 [file ijerph-18-11445-s001.zip › ijerph-1406783-supplementary.pdf]

International Journal of
Environmental Research and Public Health

Supplementary Material

The Association between Second-Hand Tobacco Smoke Exposure and Cardiorespiratory Fitness, Physical Activity, and Respiratory Health in Children

S1. Research Timeline

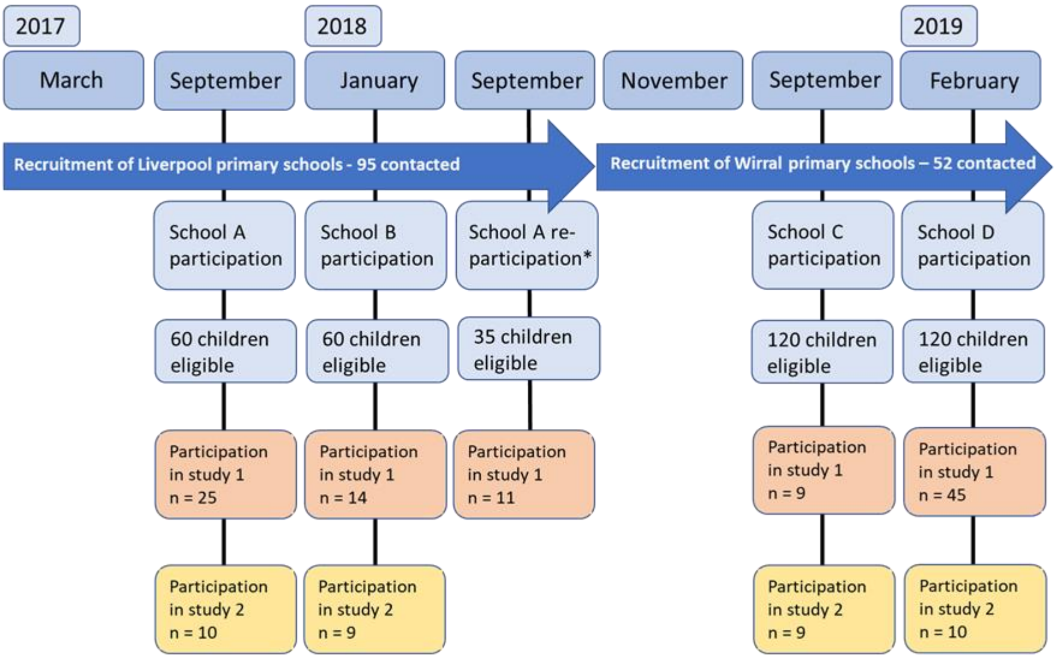

**School A participated again the following year with a new year group.*

Figure S1. Research timeline, showing recruitment, school participation, and participant selection over time.

S2. Descriptive statistics by sex

Table S2. Descriptive statistics for the sample by sex.

	Boys			Girls			T-test
	**N**	**Mean**	**SD**	**N**	**Mean**	**SD**	***p***
**Anthropometry**							
Decimal age (years)	46	10.1	0.5	58	10.2	0.7	0.810
Maturation	45	-3.1	0.6	58	-1.5	0.7	**<0.001**
Stature (cm)	45	140.1	5.8	58	142.3	7.1	0.255
Mass (kg)	45	37.4	9.1	58	38.9	9.2	0.418
BMI ($kg \cdot m^{-2}$)	45	18.7	3.7	58	19.1	4.2	0.593
**Cardiorespiratory fitness**							
VO_{2peak} ($mL \cdot min^{-1}$)	43	1736.7	285.5	51	1594.4	313.7	**0.025**
VO_{2peak} ($mL \cdot kg^{-1} \cdot min^{-1}$)	43	47.7	8.5	51	42.7	6.1	**0.002**
VO_{2peak} ($mL \cdot kg^{-0.53} \cdot min^{-1}$)	43	261.0	35.7	51	235.6	32.9	**<0.001**
**Respiratory measures**							
FEV_1 (%)	45	85.1	16.3	58	81.3	17.9	0.259

FVC (%)	45	89.4	21.3	58	88.8	18.5	0.876
PEF (%)	45	73.0	20.1	58	77.1	21.6	0.322
FEV_1/FVC	45	89.7	11.1	58	89.6	11.0	0.968
FeNO	45	17.4*	41.3†	57	14.9*	31.5†	0.317
**Children's survey**							
Physical activity	46	3.8	0.6	57	3.5	0.7	0.099
PACES	46	4.2	0.6	57	4.1	0.8	0.411
**SHS exposure**							
eCO (ppm)	43	1.7	1.2	57	1.8	1.2	0.570
Cigarettes per day	46	6.3	11.6	58	4.9	10.2	0.522
**Deprivation**							
EIMD rank	46	1481*	5400†	58	1442*	5921†	0.967

Spirometry values expressed as percentage of predicted values for sex, age, ethnicity, and height: FEV_1 = forced expiratory volume in 1 second, FVC = forced vital capacity, PEF = peak expiratory flow, FeNO = fractional exhaled nitric oxide, eCO = exhaled carbon monoxide, EIMD = English indices of multiple deprivation. Physical activity and enjoyment are scored between 1 and 5, with 5 being the most active and most enjoyment. Values which are statistically significant are highlighted in **bold.** *geometric mean, † geometric SD.

S3. Descriptive statistics by household smoking status and sex

Table S3. Descriptive statistics by household smoking status and sex.

	Non-smoking household						Smoking household						T-test (p) Non-smoking vs smoking		
	All		Boys		Girls		All		Boys		Girls		*Boys*	*Girls*	All
	N	Mean (SD)	N	Mean (SD)	N	Mean (SD)	N	Mean (SD)	N	Mean (SD)	N	Mean (SD)			
**Anthropometrics**															
Decimal age (years)	66	10.1 (0.7)	28	10.2 (0.6)	38	10.1 (0.7)	37	10.2 (0.5)	17	10.1 (0.3)	20	10.3 (0.6)	0.533	0.370	0.710
Maturation	66	-2.2 (1.1)	28	-3.2 (0.5)	38	-1.5 (0.7)	37	-2.1 (1.0)	17	-3.0 (0.8)	20	-1.5 (0.6)	0.285	0.936	0.808
Stature (cm)	66	141.6 (7.2)	28	140.8 (6.3)	38	142.1 (7.8)	37	141.9 (5.5)	17	140.9 (5.1)	20	142.8 (5.8)	0.952	0.759	0.807
Mass (kg)	66	36.4 (7.9)	28	34.9 (6.5)	38	37.5 (8.8)	37	41.6 (10.4)	17	41.3 (11.3)	20	41.6 (9.7)	**0.018**	0.106	**0.006**
BMI ($kg \cdot m^{-2}$)	66	18.1 (3.3)	28	17.5 (2.3)	38	18.5 (3.9)	37	19.0 (4.5)	17	20.7 (4.6)	20	20.4 (4.5)	**0.015**	0.103	**0.005**
**Cardiorespiratory fitness**															
VO_{2peak} ($mL \cdot min^{-1}$)	57	1645.8 (307.5)	26	1736.9 (259.3)	31	1569.5 (327.5)	37	1680.5 (311.5)	17	1736.2 (330.0)	20	1633.1 (295.0)	0.994	0.485	0.597
VO_{2peak} ($mL \cdot kg^{-1} \cdot min^{-1}$)	57	47.1 (7.1)	26	50.5 (6.9)	31	44.2 (5.8)	37	41.7 (7.6)	17	43.3 (9.1)	20	40.4 (6.0)	**0.005**	**0.028**	**0.001**
VO_{2peak} ($mL \cdot kg^{-0.53} \cdot min^{-1}$)	57	252.6 (36.7)	26	269.6 (30.7)	31	238.5 (35.6)	37	238.9 (34.5)	17	248.0 (39.5)	20	231.1 (28.4)	0.051	0.442	0.071
**Respiratory measures**															

FEV_1 (%)	66	83.3 (16.7)	28	85.7 (16.9)	38	81.5 (16.6)	37	85.9 (20.9)	17	84.2 (15.6)	20	80.9 (20.5)	0.775	0.906	0.814
FVC (%)	66	90.8 (18.9)	28	91.0 (21.5)	38	90.6 (17.0)	37	84.5 (20.0)	17	86.7 (21.4)	20	85.2 (21.1)	0.516	0.293	0.227
PEF (%)	66	76.1 (22.7)	28	73.9 (23.1)	38	77.8 (22.5)	37	73.9 (17.7)	17	71.6 (14.2)	20	75.9 (20.4)	0.720	0.749	0.609
FEV_1/FVC	66	88.3 (11.7)	28	88.5 (12.5)	38	88.1 (11.2)	37	92.0 (9.3)	17	91.2 (8.9)	20	92.4 (10.2)	0.370	0.160	0.078
FeNO (ppb)*	64	16.2 (37.1)	27	17.2 (40.2)	37	15.5 (34.8)	38	15.5 (29.3)	18	17.6 (44.0)	20	13.8 (26.3)	0.919	0.550	0.782
**Children's survey**															
Physical activity	66	3.7 (0.7)	28	3.8 (0.6)	38	3.5 (0.7)	37	3.6 (0.7)	18	3.7 (0.7)	19	3.5 (0.8)	0.400	0.912	0.604
**SHS exposure**															
eCO (ppm)	63	1.7 (1.1)	26	1.7 (1.4)	36	1.6 (0.9)	37	2.0 (1.3)	17	1.7 (0.9)	20	2.2 (1.6)	0.832	0.200	0.215
Cigarettes per day	66	0	28	0	38	0	38	15.2 (13.2)	18	16.1 (13.7)	20	14.3 (13.1)	NA	NA	NA
**Deprivation**															
EIMD rank*	66	1956 (7975)	28	1946 (7376)	38	1964 (8578)	38	879 (2709)	18	991 (3049)	20	791 (2473)	0.088	**0.044**	**0.008**

Spirometry values expressed as percentage of predicted values for sex, age, ethnicity, and height: FEV_1 = forced expiratory volume in 1 second, FVC = forced vital capacity, PEF = peak expiratory flow, FeNO = fractional exhaled nitric oxide, eCO = exhaled carbon monoxide, EIMD = English indices of multiple deprivation. Physical activity and enjoyment are scored between 1 and 5, with 5 being the most active and most enjoyment. Values which are statistically significant are highlighted in **bold**. *Indicates geometric mean and geometric standard deviation.

S4. Linear Regression for Absolute $\dot{V}O_{2peak}$

A multiple regression was run to predict absolute $\dot{V}O_{2peak}$ ($mL \cdot min^{-1}$) from the number of cigarettes smoked per day (sqrt-cigarettes), sex, age, mass, stature, maturation, PA, and logEIMD. See Table S4 the full details of the unadjusted and adjusted models for absolute $\dot{V}O_{2peak}$. Sqrt-cigarettes was not a significant predictor in the unadjusted model (R^2 = 0.001, $F(1,87)$ = 0.1, p = 0.741; adjusted R^2 = -0.01). In the adjusted model, sex, mass, stature, and PA were significant predictors whereas sqrt-cigarettes, age, maturation, and logEIMD were not. Overall, the adjusted model significantly predicted absolute $\dot{V}O_{2peak}$, and had a high R^2 value, accounting for 70.0% of the variance (R^2 = 0.728, $F(8,80)$ = 26.7, p < 0.001; adjusted R^2 = 0.700), although sqrt-cigarettes was not a significant predictor (p = 0.090) at the 0.05 level.

Table S4. Linear regression for absolute $\dot{V}O_{2peak}$ ($mL \cdot min^{-1}$).

Predictor	Unstandardised coefficient (B)	95% Confidence interval Lower bound	Upper bound	Standard error of B	Significance
	**Model 1**	**R^2 = 0.001, p = 0.741, F = 0.1**			
**Constant**	**1661.3**	1580.8	1741.9	40.5	**<0.001**
Sqrt-cigarettes	5.8	-29.2	40.8	17.6	0.741
	**Model 2**	R^2 = 0.728, p **< 0.001**, F = 26.7			
Constant	-964.2	-2218.3	289.9	630.2	0.130
Sqrt-cigarettes	-17.4	-37.5	2.8	10.1	0.091
Sex	-322.4	-465.0	-179.7	71.7	**<0.001**
Decimal age (yrs)	13.8	-57.7	85.4	36.0	0.701
Mass (kg)	23.3	17.7	28.8	2.8	**<0.001**
Stature (cm)	11.0	3.8	18.2	3.6	**0.003**
Maturation	69.1	-8.0	146.1	38.7	0.078
PA	75.6	21.2	130.1	27.4	**0.007**
LogEIMD	47.4	-15.7	110.5	31.7	0.139

S5. Household Smoking and Socioeconomic Status

Mean logEIMD was statistically higher in non-smoking households (t (102) = 2.7, p = 0.008) indicating an association between deprivation and smoking. The percentage of smoking households decreases as parental educational attainment increases, however a slight increase can be seen at 'Masters or above'. Chi-squared analysis showed the association to be almost significant at the 0.05 level (p = 0.058). Correlation analysis showed a weak statistically significant negative correlation between the square root transformed number of cigarettes smoked per day (sqrt-cigarettes) and educational attainment level (Spearman's rho r = -0.283, p = 0.006) and sqrt-cigarettes and logEIMD (Pearson r = -0.204, p = 0.038).

S6. Household Smoking and Weight Status

Mean BMI was significantly different for boys (t (43) -3.1, p = 0.015) but not girls (t (56) = -1.7, p = 0.103) from smoking and non-smoking households (Table S3). The proportion of children classed as overweight or obese varied between smoking status

groups, with children from non-smoking households most likely to have a healthy BMI. The proportion of children from smoking households who were overweight or obese was more than double (54.1%) that of non-smoking households, which was found to be statistically significant (Chi-Square (1) = 9.3, p = 0.002). There was a weak but significant positive correlation between sqrt-cigarettes and BMI (r = 0.225, p – 0.023), which was not significant when split by sex (r = 0.276, p = 0.067 for boys, r = 0.194, p = 0.145 for girls).

S7. Supplementary Data for Cardiorespiratory Fitness and Second-hand Smoke Exposure

As shown in Table S3, absolute $\dot{V}O_{2peak}$ was not found to be significantly different between children of non-smoking and smoking households (t (92) = -0.5, p = 0.597), or when boys (t (41) = 0.01, p = 0.994) and girls (t (49) = -0.7, p = 0.485) were analysed separately. Ratio scaled $\dot{V}O_{2peak}$ was significantly different between children from smoking and non-smoking homes (t (92) = 3.5, p = 0.001), and for boys (t (41) = 3.0, p = 0.005) and girls (t (49) = 2.3, p = 0.028) separately. Allometrically scaled $\dot{V}O_{2peak}$ was not statistically different between household smoking status (t (92) = 1.8, p = 0.071), but was almost statistically significant for boys (t (41) = 2.0, p = 0.051), but not for girls (t (49) = 0.8, p = 0.442).

The number of children classified as fit according to established thresholds was significantly different between SHS exposure groups (Chi-square (1) = 7.0, p = 0.008), with 91.2% and 70.3% of children from non-smoking and smoking homes, respectively, classified as fit. When split by sex, the difference remained significant for boys, of which 92.3% and 41.2% of boys from non-smoking and smoking households, respectively, were classified as fit (Chi-square (1) = 7.0, p = 0.008). For girls from non-smoking and smoking homes, 90.3% and 80.0% respectively, were classified as fit but the difference was not significant (Chi-square (1) = 1.1, p = 0.296).

Mean CRF was compared for where smoking was permitted including inside and outside the home. No significant differences in absolute and allometrically scaled fitness were observed between exposure groups, but mean ratio scaled $\dot{V}O_{2peak}$ was significantly different between groups (ANOVA (2,91) = 5.5, p = 0.005). A Tukey post hoc test showed significant differences between the 'no smoking' group and both 'inside' and 'outside' groups (p = 0.015 and 0.041, respectively), but the 'inside' and 'outside' groups were not statistically different (p = 0.665). When split by sex, significant differences were observed between mean ratio scaled $\dot{V}O_{2peak}$ and where smoking is permitted for boys (ANOVA (2,40) = 4.8, p = 0.013) but not girls, which indicated significant differences between the 'outside' and 'no smoking' groups. However, when split by where smoking is permitted, and sex, groups sizes become as small as n=7.

S8. Supplementary Data for Physical Activity and Second-hand Smoke Exposure

Mean PA (S3) was not significantly different between children of non-smoking and smoking households (t (101) = 0.5. The proportion of children classified as physically active was 87.0% for children from non-smoking homes, and 86.4% for children from smoking homes, which was not statistically significant (chi-square (1) = 0.04, p = 0.838). PA was also not significantly correlated with any smoking exposure measures including sqrt-cigarettes and eCO. PA was not significantly different between groups of where smoking is permitted in and around the home (ANOVA (2,100) = 0.7, p = 0.517)

S9. Supplementary Data for Spirometry and Household Smoking Status

No significant differences were observed for mean spirometry values for household smoking status (S4), and there were no significant correlations between sqrt-cigarettes and any spirometry measures. However, PEF% was moderately and negatively correlated with eCO (r = -0.302, p = 0.002).

When spirometry values were compared for where smoking was permitted, significant differences were observed for FER only (Table S9). A Tukey post-hoc analysis showed a significant difference between the 'no smoking' and 'outside' groups only (p = 0.038).

Table S9. Comparison of mean values for four spirometry measures for where smoking is permitted in and around the home.

Spirometry measure	Mean (±SD) No smoking	Outside only	Inside	ANOVA (2,100)	Significance p
**FEV1%**	**83.9 (17.1)**	82.0 (20.1)	80.8 (10.8)	0.2	0.782
**FVC%**	92.1 (18.7)	84.7 (22.6)	84.7 (15.7)	1.8	0.172
**PEF%**	75.8 (22.3)	78.7 (20.6)	66.1 (12.6)	1.8	0.176
**FER**	87.8 (8.1)	93.7 (8.1)	89.0 (11.0)	3.1	**0.049**

S10. Supplementary Data for Fractional Exhaled Nitric Oxide and Second-hand Smoke Exposure

Mean logFeNO was not significantly different between children from smoking and non-smoking households (S4) and logFeNO was not correlated with the number of cigarettes smoked per day.

Mean FeNO was lower for children from households where smoking is permitted inside (13.5 ± 12.2 ppb) compared to smoking outside (21.6 ± 26.4 ppb) and no smoking (22.9 ± 23.6 ppb) although the difference was not significant (ANOVA (2, 95) = 2.0, p = 0.135).

S11. Supplementary Data for Linear Regressions for Spirometry Measures

Table S11.1. Linear regressions for spirometry measure FEV1%.

	Unstandardised coefficient (B)	95% Confidence interval Lower bound	Upper bound	Standard error of B	Significance
	**Model 1**	**$R^2 < 0.001$, $p = 0.864$, $F = 0.03$**			
**Constant**	**83.2**	79.0	87.3	2.1	**<0.001**
Sqrt-cigarettes	-0.2	-2.0	1.7	0.9	0.864
	**Model 2**	$R^2 = 0.138$, $p =$ **0.005**, F = 3.9			
Constant	48.1	23.0	73.3	12.7	**<0.001**
Sqrt-cigarettes	0.4	-1.4	2.2	0.9	0.660
Mass (kg)	0.0	-0.4	0.4	0.2	0.964
Asthma	6.6	-4.7	17.9	5.7	0.250
LogEIMD	10.6	5.0	16.2	2.8	**<0.001**

Table S11. 2. Linear regressions for spirometry measure FVC%.

	Unstandardised coefficient (B)	95% Confidence interval Lower bound	Upper bound	Standard error of B	Significance
	**Model 1**	**$R^2 = 0.013$, $p = 0.247$, $F = 1.4$**			
**Constant**	**90.6**	85.9	95.3	2.4	**<0.001**
Sqrt-cigarettes	-1.2	-3.3	0.9	1.1	0.247
	**Model 2**	$R^2 = 0.135$, $p =$ **0.006**, F = 3.8			
Constant	47.2	18.4	76.0	14.5	0.002
Sqrt-cigarettes	-0.7	-2.8	1.3	1.0	0.494
Mass (kg)	0.1	-0.3	0.5	0.2	0.542
Asthma	4.6	-8.4	17.5	6.5	0.486
LogEIMD	11.8	5.4	18.2	3.2	**<0.001**

Table S11. 3. Linear regressions for spirometry measure PEF%.

	Unstandardised coefficient (B)	95% Confidence interval Lower bound	Upper bound	Standard error of B	Significance
	**Model 1**	**$R^2 = 0.002$, $p = 0.659$, $F = 0.2$**			
**Constant**	**75.4**	70.6	80.2	2.4	**<0.001**
Sqrt-cigarettes	-0.6	-2.7	1.6	1.1	0.608
	**Model 2**	$R^2 = 0.064$, $p = 0.166$, F = 1.7			
Constant	66.8	37.1	96.5	15.0	**<0.001**
Sqrt-cigarettes	0.0	-2.2	2.1	1.1	0.965
Mass (kg)	-0.2	-0.6	0.2	0.2	0.352
Asthma	12.4	-1.0	25.7	6.7	0.070
LogEIMD	4.6	-2.0	11.2	3.3	0.171

Table S11. 4. Linear regressions for spirometry measure FER.

	Unstandardised coefficient (B)	95% Confidence interval		Standard error of B	Significance
		Lower bound	Upper bound		
	**Model 1**	**R^2 = 0.015, *p* = 0.227, *F* = 1.5**			
**Constant**	**89.5**	87.0	91.8	1.2	**<0.001**
Sqrt-cigarettes	0.6	-0.4	1.7	0.5	0.227
	**Model 2**	R^2 = 0.030, *p* = 0.561, F = 0.7			
Constant	81.3	66.0	96.7	7.7	**<0.001**
Sqrt-cigarettes	0.7	-0.4	1.8	0.5	0.204
Mass (kg)	0.1	-0.2	0.3	0.1	0.645
Asthma	-1.5	-8.4	5.3	3.5	0.658
LogEIMD	2.0	-1.5	5.4	1.7	0.260

S12. Exhaled Carbon Monoxide and Household Smoking

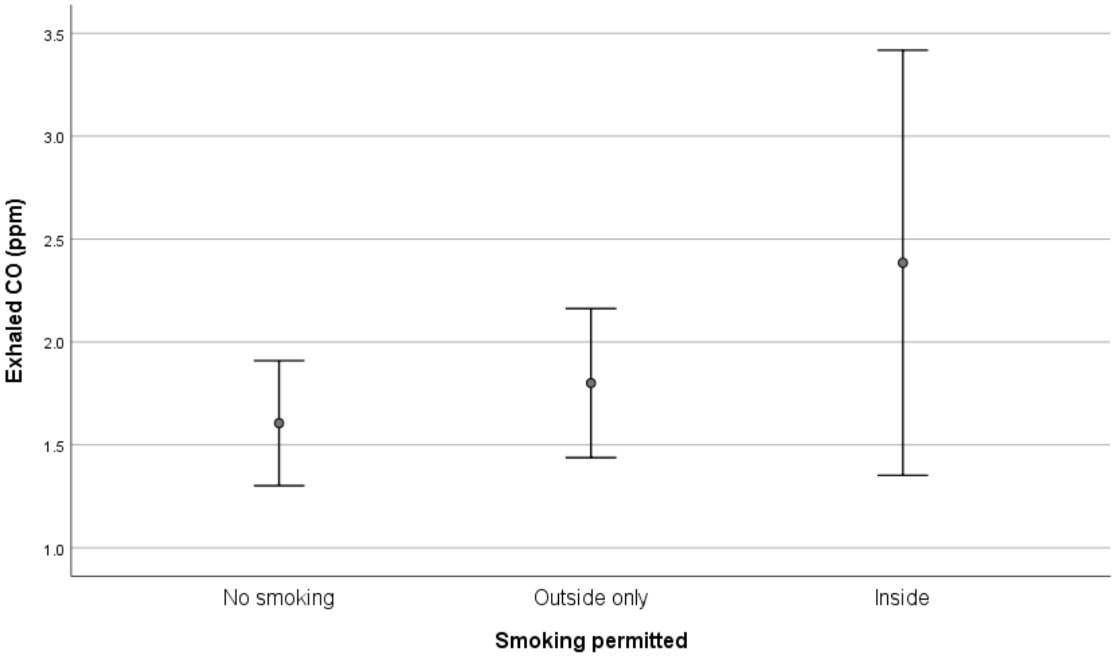

Figure S12. Mean exhaled CO (ppm) with 95% confidence per where smoking is permi.
